# Histiocytose langerhansienne cérébrale

**DOI:** 10.11604/pamj.2015.22.345.8401

**Published:** 2015-12-10

**Authors:** Assou Ajja, Rachid Ammor

**Affiliations:** 1 Neurosurgery, Military Hospital My Ismail, Meknes, Morocco

**Keywords:** Tumeur cérébrale, chirurgie, histiocytose langerhansienne, brain tumor, surgery, Langerhans cell histiocytosis

## Image en medicine

Un patient de 19 ans, sans antécédents particuliers, admis pour des crises convulsives généralisées depuis un mois. L'examen clinique initial était sans particularités. L'IRM cérébrale a objectivé deux lésions temporales gauches intra axiales à double composante kystique et charnue avec prise de contraste intense en périphérie. Le patient a bénéficié d'une exérèse tumorale à visée diagnostique et thérapeutique. L'examen anatomopathologique a posé le diagnostic d'une histiocytose cérébrale. Le bilan d'extension de la maladie comportant notamment une scintigraphie osseuse, un scanner thoraco-abdomino-pelvien était sans particularité. L'histiocytose langerhansienne est une pathologie à développement multi viscéral potentiel liée à la prolifération et à l'accumulation au sein de certains tissus de cellules de langerhans. Son diagnostic est anatomopathologique reposant sur la mise en évidence par immunohistochimie de l'expression cellulaire de la protéine S100 et l'antigène CD1a, et la présence de granules de Birbeck en microscopie électronique. Cette pathologie atteint préférentiellement l'os, la peau, les poumons, le foie ou encore le sang. La localisation cérébrale reste exceptionnelle.

**Figure 1:**
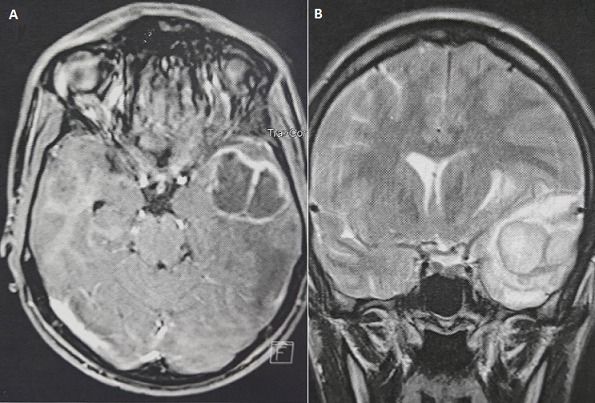
A) IRM cérébrale en séquence pondérée T1 injectée en coupe axiale montrant un processus intra axial temporal gauche à double composante charnue et kystique de 40×44mm, qui se rehausse en périphérie après injection de produit de contraste, avec œdème péri lésionnel en doigt de gant; B) IRM cérébrale en séquence pondérée T2 en coupe coronale montrant l'effet de masse sur les structures médianes et le ventricule latéral homolatéral ainsi que l’œdème en hyper signal T2

